# Polymorphisms in PCSK9, LDLR, BCMO1, SLC12A3, and KCNJ1 Are Associated with Serum Lipid Profile in Chinese Han Population

**DOI:** 10.3390/ijerph16173207

**Published:** 2019-09-02

**Authors:** Zheng Li, Tianyu Zhao, Xiaohua Tan, Song Lei, Liu Huang, Lei Yang

**Affiliations:** 1 Medical School, Hangzhou Normal University, Hangzhou 310000, China; 2 Medical School, Shihezi University, Shihezi 832000, China

**Keywords:** *PCSK9*, *LDLR*, *SLC12A3*, plasma lipid levels, dyslipidemia

## Abstract

Unfavorable serum lipid levels are the most important risk factors for coronary artery disease (CAD), cerebral infarction, and other cardiovascular and cerebrovascular diseases. This study included 2323 Han Chinese in southern China. We collected medical reports, lifestyle details, and blood samples of individuals and used the polymerase chain reaction-ligase detection reaction method to genotype single-nucleotide polymorphisms (SNPs). Two SNPs showed a strong evidence of association with total cholesterol (TC): rs1003723 and rs6413504 in the low-density lipoproteins receptor (*LDLR*). Two SNPs in *LDLR* showed a strong evidence of association with low-density lipoprotein cholesterol (LDL-C), rs1003723 and rs6413504. Two SNPs showed a strong evidence of association with triglycerides (TG), namely, rs662145 in pro-protein convertase subtilisin-kexin type 9 (*PCSK9)* and rs11643718 in the solute carrier family 12 member 3 (*SLC12A3)*. For the TC, LDL-C, and TG levels, these SNPs generated strong combined effects on these lipid levels. For each additional dangerous gene, TC increased by 0.085 mmol/L (*p* = 7.00 × 10^−6^), and LDL-C increased by 0.075 mmol/L (*p* = 9.00 × 10^−6^). The TG increased by 0.096 mmol/L (*p* = 2.90 × 10^−5^). Compared with those bearing no risk alleles, the risk of hypertriglyceridemia, hypercholesterolemia, and dyslipidemia increased in those with two or more risk alleles and one risk gene. Polymorphisms of *PCSK9*, *LDLR*, and *SLC12A3* were associated with the plasma lipid levels in people in southern China. These results provide a theoretical basis for gene screening and the prevention of dyslipidemia.

## 1. Introduction

Epidemiological studies have shown that among the risk factors of coronary artery disease (CAD), cerebral infarction, including other cardiovascular and cerebrovascular diseases, dyslipidemia is an important factor [1,2,3]. The characteristics of dyslipidemia are very complex, mainly due to genetic or environmental factors [4], as well as their interaction [5]. According to the study of twins and family history, it can be inferred that about half of the individual variation of the blood lipid phenotype is caused by genetic factors. [6,7].

According to recent research of genome-wide association studies (GWAS), a large number of single-nucleotide polymorphisms (SNPs) have been authenticated, which are related to lipid levels in Caucasian-descent populations [4,8,9,10,11,12,13,14]. These SNPs were originally identified among the ancestors of Europeans, whereas among Asian populations, especially Chinese, just a handful of genes were assessed. Whether these variants also confer the risk of dyslipidemia on the Han Chinese population, with its different demographic and lifestyles, has hardly been explored.

A lot of genetic variations in the low-density lipoproteins receptor (*LDLR*) gene are associated with increased plasma low-density lipoprotein cholesterol (LDL-C) levels [15,16,17]. Pro-protein convertase subtilisin-kexin type 9 (*PCSK9*) can affect the *LDLR* protein levels, which can lead to changes in the cholesterol metabolism and low-density lipoprotein (LDL) particle concentration in blood [15,18]. Few studies have shown that beta-carotene monooxygenase 1 (*BCMO1*), solute carrier family 12 member 3 (*SLC12A3*), and potassium voltage-gated channel subfamily J member 1 (*KCNJ1*) can influence the change of blood lipids. According to the latest research, the variations of rs6564851 in *BCMO1* are obviously related to the level of the high-density lipoprotein cholesterol (HDL-C) [19]. To our knowledge, no associations between dyslipidemia, *SLC12A3,* and *KCNJ1* have been reported.

In this study, 11 SNPs in five genes were selected to assess the relevance of these SNPs and serum lipids profiles. These SNPs are: rs2479409 and rs662145 in *PCSK9*; rs2738466, rs1003723, and rs6413504 in *LDLR*; rs11646692 and rs12934922 in *BCMO1*; rs11643718 in *SLC12A3*; and rs675759, rs675388, and rs2846679 in *KCNJ1*. Based on multiple linear regression, we got the genotypes that affect the lipid levels, then calculated the scores of the combined effects of genotypes on every lipid degree and dyslipidemia.

## 2. Materials and Methods

### 2.1. Subjects

A total of 2323 Han Chinese (1069 men and 1254 women) were recruited from the Yinzhou District of Ningbo city, Zhejiang, China. All subjects had to meet the following criteria: (1) Permanent residents aged more than 40 years old; (2) Han ethnic; (3) no consanguinity relation; (4) free from stroke, diabetes, hypertension, CAD, cerebral infarction, cancer, tuberculosis, and infectious diseases such as the acquired immune deficiency syndrome (AIDS). The individuals who are nephropathy patients or taking lipid-lowering drugs were excluded from the study. This scheme has also been given permission by the Institute Research Board of Hangzhou Normal University (No. 2013020), and informed consents were obtained from the individuals.

### 2.2. Diagnostic Criteria

Dyslipidemia was diagnosed according to the criteria set by the National Cholesterol Education Program-Adult Treatment Panel III (NCEP-ATP III) and classified into four phenotypes [20]: (1) Isolated hypertriglyceridemia: Serum triglycerides (TG) ≥ 1.7 mmol/L and total cholesterol (TC) < 6.2 mmol/L; (2) isolated hypercholesterolemia: TC ≥ 6.2 mmol/L and triglycerides < 1.7 mmol/L; (3) mixed hyperlipidemia: Triglycerides ≥ 1.7 mmol/L and TC ≥ 6.2 mmol/L; (4) isolated low HDL-C:HDL-C ≤ 1.03 mmol/L (male) and ≤ 1.29 mmol/L (female) without hypertriglyceridemia or hypercholesterolemia.

### 2.3. Epidemiological Investigation

At the time of enrollment, background data, such as gender, age, height, and body weight, were collected from each subject. 

The risk factors of dyslipidemia, including smoking, drinking status, and exercise habit, were collected with a formatted questionnaire. The primary variables of lifestyle were viewed as the following. (1) Current smoking was defined as smoking at least one cigarette a day for more than one year; (2) current drinking was defined as drinking at least 500 mL of beer or 150 mL of wine or distilled spirits a day for more than one year; (3) average weekly exercise less than three times was defined as “no exercise” and average weekly exercise three or more times was defined as “regular exercise”. 

Venous blood was collected after fasting for eight hours, and the concentration of the TG, TC, LDL-C, and HDL-C were analyzed by enzymatic methods with a commercially available kit (RANDOX Laboratories Ltd., London, England). Each measurement was performed with an autoanalyzer (Type 7170A; Hitachi Ltd., Tokyo, Japan).

### 2.4. Isolation of Genomic DNA

The genomic DNA was extracted from blood cells, which were made from centrifugal experiments and stored at minus 80 degrees Celsius, by the standard phenol/chloroform scheme. Finally, all of the genomic DNA was analyzed by electrophoresis.

### 2.5. SNP Selection and Genotyping

The 11 SNPs in five genes were selected using the HapMap website and Haploview 4.2: rs2479409 resides in the 2 kb upstream variant and rs662145 resides in the intron of *PCSK9*; rs2738466, rs1003723, and rs6413504 reside in the intron of *LDLR*; rs11646692 resides in the 2 kb upstream variant and rs12934922 resides in the missense variant of *BCMO1*; rs11643718 resides in the missense variant of *SLC12A3*; rs675759 and rs675388 reside in the 3′-utr and rs2846679 is in the intron of *KCNJ1*. The minor allele frequencies (MAFs), an uncommon allele frequency in a given population, of these SNPs were greater than 5%. In association studies, a small MAF would reduce the statistical efficiency, which may be the factors causing a false negative reaction. So MAF is usually required to be greater than 0.0.5 or 0.10. The linkage disequilibrium r^2^ is greater than 0.8, which means that these SNPs were in a strong linkage disequilibrium.

The genotypes of 11 SNPs in each subject were characterized by the PCR-ligase detection reaction (PCR-LDR) with specific primers and probes listed in Table S1. Simply speaking, the sequences containing these SNP sites were amplified by multiple PCR. Then, each SNP site was characterized for the LDR with three probes, two of which are complementary to the bases near the SNP site. Except for one base at the 3’end, the other bases are similar. The remaining probe was labelled with fluorescence and was complementary to the other end of the SNP site. Therefore, the complete complementary sequences were linked together by the catalyzing of ligase, resulting in LDR products of different lengths for different genotypes [21,22]. The results were analyzed by the Genemapper software [23]. At the same time, some PCR and LDR productions were selected randomly for further cloning and sequencing. In order to control the quality, we randomly selected 10% of the samples to re-classify the genes, and the concordance was 99.5% (rs11646692), 99.8% (rs12934922), 99.5% (rs2479409), 99.8% (rs662145), 99.4% (rs2738466), 99.4% (rs1003723), 99.4% (rs6413504), 99.6% (rs11643718), 99.7% (rs675759), 99.6% (rs675388), and 99.4% (rs2846679). Generally speaking, the success rate of classification of an SNP in all samples should be more than 75%, otherwise, the SNP cannot participate in the subsequent calculation.

### 2.6. Statistical Analysis

Continuous variables are presented as means ± SD values if the distribution is normal, and median (interquartile range) if the distribution is skewed. Categorical variables are presented as percentages (%). The effects of gene variants on lipid levels were analyzed by the multivariable linear regression analysis, and age, gender, body mass index, exercise, waist circumference, smoking, and alcohol consumption were used as covariates. In the additive model, homozygotes for the major allele, heterozygotes, and homozygotes for the minor allele were coded to an ordered categorical variable for the genotype (zero, one, and two, respectively) [24]. On the basis of the genotype-phenotype association analyses, we further performed the cumulative analysis with the lipid traits that had at least two associated SNPs. Assuming that a single SNP has the same effect on the dyslipidemia and lipid traits, the number of risk genes carried by each participant is calculated to establish a genotype score model. The cumulative effects of SNPs for the plasma TG, TC, HDL-C, and LDL-C were assessed by the multivariable linear regression. The cumulative effects of SNPs for dyslipidemia were estimated by the binary logistic regression analysis. The associations between SNP and the risk of dyslipidemia were calculated using the R language package “forestplot”. Statistical analyses were performed using the SPSS 24.0 software (SPSS Inc., Chicago, IL, USA). Two-tailed p-values of <0.05 were considered significant.

## 3. Results

### 3.1. General Characteristics, Serum Lipid Levels, and Lifestyles

In our study, a total of 2323 subjects (1069 men and 1254 women) were recruited, and a summary of demographic characteristics such as age, BMI, and waist circumference, and the levels of TC, HDL-C, and LDL-C are provided in Table 1. The median age was 59 (49–67) years and the median BMI was 23.00 (21.12~25.11) kg/m^2^ in total. People who always smoke and drink account for 26.30% and 31.47% of the total number, respectively. The minor allele frequency (MAF) of each SNP was more than 5% to ensure that this study had enough statistical power (Table 2). The significance of the Hardy–Weinberg equilibrium (HWE) lies in testing the results of the sampling survey, evaluating whether the group in the study conforms to the HWE, and then evaluating the reliability of the survey data. P > 0.05 indicates that the investigated population has reached the genetic equilibrium, which means that the data of this population survey are reliable. Ten of the eleven SNPs were in the Hardy–Weinberg equilibrium (*p* > 0.05) in this research.

### 3.2. SNPs Associated with TC, HDL-C, LDL-C, and TG

Table 3 shows the association between each of the 10 SNPs with levels of TC, TG, HDL-C, and LDL-C by the multiple linear regression analyses. After adjustment for age, gender, BMI, waist circumference, smoking, drinking, and exercise, two SNPs showed a strong evidence of association with total cholesterol: rs1003723 and rs6413504 in *LDLR* (0.15 mmol/L per T, *p* = 6.80 × 10^−^^5^ and 0.11 mmol/L per G allele, *p* = 1.45 × 10^−^^4^, respectively). No variants showed a strong evidence of association with HDL-C. Two SNPs in *LDLR* also showed a strong evidence of association with LDL-C, rs1003723 in *LDLR* (increase 0.13 mmol/L per T allele, *p* = 8.70 × 10^−^^5^), rs6413504 in *LDLR* (increase 0.10 mmol/L per G allele, *p* = 1.55 × 10^−^^4^). Two SNPs showed a strong evidence of association with TG, namely, rs662145 in *PCSK9* (0.09 mmol/L per T allele, *p* = 7.64 × 10^−^^4^) and rs11643718 in *SLC12A3* (0.11 mmol/L per A allele, *p* = 1.00 × 10^−^^3^) (shown in Table 3). Following a Bonferroni correction [25], the above results are still statistically significant.

### 3.3. Combined Effects of Genetic Variants on Lipid Levels and Dyslipidemia

We then defined the alleles with the effect of increasing TC, LDL-C, or TG as the risk allele; an individual may carry zero, one, or two risk alleles for each SNP. We calculated the risk allele numbers for each subject to a genotype score of TC, LDL-C, and TG. The association of the genotype score and its corresponding lipid levels were evaluated by the multiple linear regression analyses after adjustment for age, gender, BMI, waist circumference, and lifestyle data. As shown in Table 4, the mean TC, LDL-C, and TG levels increased with the genotype score. The TC level increased from 4.83 mmol/L for those without risk alleles to 5.34 mmol/L for those with 4 risk alleles. The TC increased by 0.085 mmol/L for each additional dangerous gene (*p* for trend, 7.00 × 10^−6^). The LDL-C level increased from 3.01 mmol/L for those with no risk allele to 3.41 mmol/L for those with four risk alleles. The LDL-C increased by 0.075 mmol/L for each additional dangerous gene (*p* for trend, 9.00 × 10^−6^). The TG level increased from 1.23 mmol/L for those without the risk allele to 1.31 mmol/L for those with four risk alleles. The TG increased by 0.096 mmol/L for each additional dangerous gene (*p* for trend, 2.90 × 10^−5^) (Table 4).

**Figure 1:**
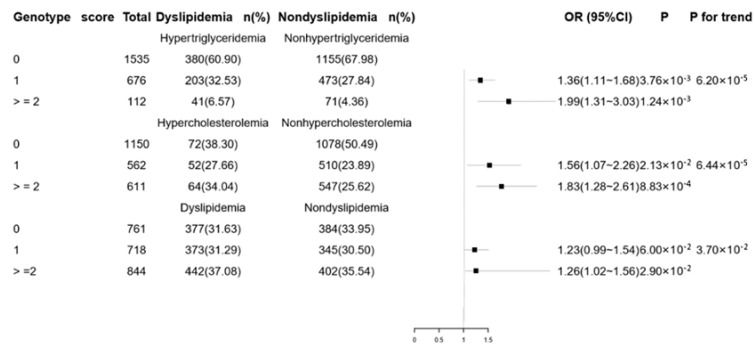
Cumulative effects of the risk alleles on dyslipidemia. Number of risk alleles for hypertriglyceridemia including two single-nucleotide polymorphisms (SNPs): Rs662145 and rs11643718. Number of risk alleles for hypercholesterolemia including 2 SNPs: Rs1003723 and rs6413504. Number of risk alleles at the for dyslipidemia including 4 SNPs: Rs662145, rs11643718, rs1003723, and rs6413504. Adjusted for sex, age, BMI, waist circumference, smoking, drinking, and exercise. The above results are still statistically significant after a Bonferroni correction.

Although no single SNP was associated with dyslipidemia in this study (data not shown), we found that compared with those bearing no risk alleles, the hypertriglyceridemia risk increased in those with two or more risk alleles and one risk allele (OR = 1.99, 95% CI = (1.31~3.03), *p* = 1.24 × 10^−3^; OR = 1.36, 95% CI = 1.11~1.68, *p* = 3.76 × 10^−3^); compared with those bearing no risk allele, the hypercholesterolemia risk increased in those with two or more risk alleles and one risk allele (OR = 1.83, 95% CI = (1.28~2.61), *p* = 8.83 × 10^−4^; OR = 1.56, 95% CI = 1.07~2.26, *p* = 2.13 × 10^−2^); compared with those bearing no risk allele, the dyslipidemia risk increased in those with two or more risk alleles (OR = 1.26, 95% CI = (1.02~1.56), *p* = 2.90 × 10^−2^). Those with a higher genotype score have a higher risk of hypertriglyceridemia, hypercholesterolemia, and dyslipidemia (*p* for trend, 6.20 × 10^−5^; *p* for trend, 6.44 × 10^−4^; *p* for trend, 3.07 × 10^−2^, respectively) (Figure 1).

## 4. Discussion

In the present study, we confirmed that four SNPs in three genes were associated with the lipid level in a Chinese Han population. After adjusting the age, gender, BMI, waist circumference, and lifestyle characteristics, we found that some SNPs influenced the lipid traits, two SNPs in *LDLR* (rs1003723 and rs6413504) associated with the lipid levels, such as TC and LDL-C, and one SNP (rs662145) in *PCSK9* and one SNP (rs11643718) in *SLC12A3* were associated with the TG levels.

The *LDLR* and *PCSK9* variants have been shown to be associated with the plasma lipid, especially cholesterol metabolism [11,15,18,26,27,28]. As we know, low-density lipoprotein cholesterol receptor-related proteins (LRPs) are transmembrane receptors involved in endocytosis, cell signaling, and trafficking of other cellular proteins, which is produced in the endoplasmic reticulum of the hepatocytes, and attain maturity in the Golgi apparatus, then get expressed on the cell surface. We found that two variants of the *LDLR* strongly influenced the LDL-C and TC levels. The rs1003723 and rs6413504 in *LDLR* are mainly associated with hypercholesterolemia, which is consistent with several studies [29,30,31,32]. Both rs1003723 and rs6413504 in the *LDLR* can affect the expression of *LDLR*. This receptor binds specifically to the apolipoprotein B (ApoB) in the LDL particle on fibroblasts, and then the LDL-receptor complex is internalized by endocytosis, which is mediated by the LDL receptor adaptor protein. The LDL receptor is then recycled back to the hepatocyte, where it can again bind with ApoB particles, and the LDL particle undergoes lysosomal degradation, releasing cholesterol. So far, in the ClinVar database, nearly 1500 “pathogenic” and “likely pathogenic” variants of the *LDLR* have been listed [33]. The rs662145 in *PCSK9* is newly found to be associated with hypertriglyceridemia. The rs662145 G allele is associated with increased TG levels. The rs662145 variants in *PCSK9* may increase the expression of proprotein convertase subtilisin/kexin type 9, a protein which is expressed in the liver, intestine, and kidney tissues and escorts specific receptors for lysosomal degradation. It plays a role in fatty acid metabolism. Fatty acids are one of the important materials for synthesis of triglycerides. Therefore, mutations in this gene may increase the triglyceride levels, leading to hypertriglyceridemia. It is worth noting that the SNPs associated with TC are the same as those for LDL-C and the magnitude of the effect is almost the same. Hypercholesterolemia is caused by an excess of LDL-C, and the associations between these SNPs and TC may actually be the association between these SNPs and LDL-C.

The rs11643718 in the solute carrier family 12 member 3 (*SLC12A3*) is another newly found SNP associated with hypertriglyceridemia. The rs11643718 A allele is associated with increased TG levels. Animal studies have shown that supplementation with arginine prevented an increase in the LDL-C and TG levels [34,35]. The rs11643718 resides in the coding area of *SLC12A3*. When the G allele mutates to the A allele, the missense mutation changes the amino acid encoded by this codon from arginine to glutamine. Arginine decreased, leading to an increase of TG. In addition, the gene variants in the *SLC12A3* have been reported as a genetic susceptibility factor of cardiovascular diseases such as type 2 diabetes [36]. However, such studies are rare, and more studies are needed to prove the association of the *SLC12A3* with lipid-related diseases.

Quite different from the other studies, the genes we finally chose to study were those that don’t have enough evidence to show that they can affect the plasma lipid levels in the past research. Beta-carotene monooxygenase 1 (*BCMO1*) catalyzes the first step in the central cleavage and conversion of dietary provitamin carotenoids to vitamin A (retinal) in the small intestine [37]. In a recent study, the rs6564851 in *BCMO1* was significantly associated with the HDL-C levels [19]. Potassium inwardly rectifying channel, subfamily J, member 1 (*KCNJ1*) gene, which is located at chromosome 11p24, encodes a protein for a voltage-gated potassium channel. Until now, to our knowledge, no exact evidence has been provided of the effect of *KCNJ1* on the plasma lipid levels. However, a recent study showed that another member belongs to the potassium inwardly rectifying channel family, *KCNQ1*, and its genetic variants are associated with the TG and HDL levels [38]. In our study, we found no such meaningful results of *BCMO1* and *KCNJ1*. The effect of *BCMO1* and *KCNJ1* on the plasma lipid levels and mechanisms needs further studies.

In this study, we identified two SNPs associated with the TC levels, two SNPs associated with the LDL-C levels, two SNPs associated with the TG levels, and four SNPs associated with dyslipidemia, such as hypercholesterolemia and hypertriglyceridemia. The cumulative effect of the risk allele numbers in these SNPs and lipid levels and various types of dyslipidemia were analyzed. Those with more risk allele numbers have higher lipid levels and dyslipidemia risk.

This study has some limitations. First, it was carried out only in Ningbo. Due to geographical restrictions, whether our findings can be applied to other groups remains to be explored. Second, most of the questions in the epidemiological questionnaire were obtained by asking, and there may be a recall bias. Third, when scoring risk genes, we assumed that each gene had the same risk and ignored the actual effect of each gene on the lipid levels and dyslipidemia.

## 5. Conclusions

In summary, we identified a set of SNPs associated with lipid levels in a Chinese Han population. Our results suggest that PCSK9, LDLR, and SLC12A3 may be involved in the plasma lipid and lipoprotein metabolism. Additionally, we further identified variants that showed combined effects on the lipid levels. Those with more risk allele numbers have higher lipid levels and higher dyslipidemia risk.

## Figures and Tables

**Table 1:** General characteristics, serum lipid levels, and lifestyles.

Charecteristics	Male	Female	Total
	1069	1254	*n* = 2323
Age (years)	60 (50~69)	58 (49~65)	59 (49–67)
BMI (kg/m^2^)	22.85 (21.07~24.92)	23.17 (21.17~25.23)	23.00 (21.12~25.11)
Waist circumference (cm)	82 (77~88)	80 (75~86)	81 (76–87)
TC (mmol/l)	4.78 ± 0.90	4.98 ± 0.93	4.89 ± 0.92
TG (mmol/l)	1.20 (0.92~1.63)	1.33 (0.99~1.86)	1.28 (0.96~1.76)
HDL-C (mmol/l)	1.30 ± 0.34	1.29 ± 0.28	1.29 ± 0.31
LDL-C (mmol/l)	2.98 ± 0.78	3.13 ± 0.84	3.06 ± 0.81
SBP	134.64 ± 19.20	133.92 ± 19.13	134.25 ± 19.16
DBP	82.66 ± 12.02	81.05 ± 11.86	81.79 ± 11.96
Current smoking *n* (%)	588 (55.00)	23 (1.83)	611 (26.30)
Current drinking *n* (%)	592 (55.38)	139 (11.08)	731 (31.47)
Exercise *n* (%)			
No	729 (68.19)	764 (60.92)	1493 (64.27)
Regular	340 (31.81)	490 (39.07)	830 (35.73)

BMI: Body mass index; TC: Total cholesterol; TG: Triglyceride; HDL-C: High-density lipoprotein cholesterol; LDL-C: Low-density lipoprotein cholesterol; SBP: Systolic blood pressure; DBP: Diastolic blood pressure.

**Table 2:** Test of the Hardy–Weinberg equilibrium and allele frequency.

SNP	Gene	Chromosome	Major Allele	Minor Allele	MAF ^a^ (%)	*HWE-p* Value
rs11646692	*BCMO1*	16	C	G	34.22	0.037
rs12934922	*BCMO1*	16	A	T	13.53	0.875
rs2479409	*PCSK9*	1	A	G	31.62	0.217
rs662145	*PCSK9*	1	C	T	10.70	0.472
rs2738466	*LDLR*	19	A	G	38.92	0.976
rs1003723	*LDLR*	19	C	T	14.82	0.345
rs6413504	*LDLR*	19	A	G	27.26	0.454
rs11643718	*SLC12A3*	16	G	A	8.79	0.251
rs675759	*KCNJ1*	11	C	G	12.52	0.921
rs675388	*KCNJ1*	11	T	A	11.70	0.804
rs2846679	*KCNJ1*	11	G	A	10.96	0.392

^a^ MAF: Minor allele frequency.

**Table 3:** Association of the ten SNPs with TG, TC, HDL-C, and LDL-C.

Polymorphisms		TC mmol/L	HDL-C mmol/L	LDL-C mmol/L	TG mmol/L
*BCMO1*	AA (n = 1742)	4.87 ± 0.90	1.30 ± 0.31	3.05 ± 0.80	1.26 (0.95~1.75)
rs12934922	AT (n = 537)	4.94 ± 0.99	1.29 ± 0.29	3.09 ± 0.84	1.31 (0.97~1.85)
	TT (n = 44)	4.88 ± 1.03	1.34 ± 0.29	3.08 ± 0.92	1.23 (0.94~1.69)
	effect ^a^	0.06 (−0.02~0.13)	−0.01 (−0.03~0.02)	0.04 (−0.02~0.11)	0.06 (0.01~0.12)
	*p*	1.42 × 10^−1^	5.84 × 10^−1^	1.99 × 10^−1^	3.20 × 10^−2^
*PCSK9*	AA (*n* = 1074)	4.85 ± 0.89	1.29 ± 0.30	3.04 ± 0.79	1.27 (0.96~1.73)
rs2479409	AG (*n* = 1026)	4.93 ± 0.95	1.30 ± 0.33	3.09 ± 0.83	1.28 (0.95~1.77)
	GG (*n* = 223)	4.89 ± 0.95	1.27 ± 0.29	3.02 ± 0.84	1.29 (0.95~1.84)
	effect ^a^	0.04 (−0.02~0.10)	0.00 (−0.02~0.02)	0.01 (−0.04~0.06)	0.01 (−0.03~0.06)
	*p*	1.67 × 10^−1^	9.42 × 10^−1^	7.01 × 10^−1^	4.93×10^−1^
*PCSK9*	CC (*n* = 1851)	4.88 ± 0.92	1.30 ± 0.31	3.06 ± 0.83	1.26 (0.95~1.72)
rs662145	CT (*n* = 441)	4.94 ± 0.92	1.28 ± 0.33	3.07 ± 0.75	1.36 (1.00~1.92)
	TT (*n* = 31)	4.92 ± 0.87	1.24 ± 0.22	3.27 ± 0.90	1.23 (0.86~2.13)
	effect ^a^	0.06 (−0.03~0.14)	−0.02 (−0.05~0.01)	0.03 (−0.04~0.11)	0.09 (0.03~0.15)
	*p*	1.89 × 10^−1^	1.91 × 10^−1^	3.57 × 10^−1^	**7.64 × 10**^−**4**^
*LDLR*	AA (*n* = 871)	4.87 ± 0.90	1.30 ± 0.31	3.05 ± 0.80	1.26 (0.96~1.72)
rs2738466	AG (*n* = 1102)	4.92 ± 0.92	1.30 ± 0.32	3.08 ± 0.82	1.28 (0.95~1.75)
	GG (*n* = 350)	4.86 ± 0.96	1.25 ± 0.28	3.05 ± 0.82	1.30 (0.97~1.87)
	effect ^a^	0.01 (−0.06~0.05)	−0.02 (−0.04~0.00)	0.00 (−0.05~0.05)	0.01 (−0.03~0.05)
	*p*	9.04 × 10^−1^	4.80 × 10^−2^	9.79 × 10^−1^	5.08×10^−1^
*LDLR*	CC (*n* = 1694)	4.85 ± 0.90	1.30 ± 0.31	3.03 ± 0.79	1.28 (0.96~1.74)
rs1003723	CT (*n* = 574)	5.00 ± 0.98	1.31 ± 0.33	3.16 ± 0.87	1.28 (0.94~1.86)
	TT (*n* = 55)	4.99 ± 0.98	1.35 ± 0.26	3.18 ± 0.86	1.24 (0.91~1.79)
	effect ^a^	0.15 (0.08~0.22)	0.02(−0.01~0.04)	0.13 (0.06~0.19)	0.02 (−0.03~0.08)
	*p*	**6.80 × 10**^−**5**^	1.91 × 10^−1^	**8.70 × 10**^−**5**^	4.09×10^−1^
*LDLR*	AA (*n* = 1234)	4.83 ± 0.90	1.29 ± 0.31	3.00 ± 0.77	1.28 (0.96~1.74)
rs6413504	AG (*n* = 913)	4.95 ± 0.95	1.29 ± 0.31	3.11 ± 0.86	1.28 (0.95~1.79)
	GG (*n* = 176)	4.59 ± 0.85	1.33 ± 0.32	3.17 ± 0.87	1.29 (0.92~1.85)
	effect ^a^	0.11 (0.05~0.17)	0.01 (−0.01~0.03)	0.10 (0.05~0.15)	0.01 (−0.03~0.06)
	*p*	**1.45 × 10**^−**4**^	5.14 × 10^−1^	**1.55 × 10**^−**4**^	5.45×10^−1^
*SLC12A3*	GG (*n* = 1930)	4.89 ± 0.91	1.30 ± 0.31	3.08 ± 0.81	1.26 (0.95~1.72)
rs11643718	GA (*n* = 379)	4.88 ± 0.93	1.28 ± 0.31	3.00 ± 0.83	1.35 (0.99~1.93)
	AA (*n* = 14)	4.90 ± 0.98	1.31 ± 0.25	2.59 ± 0.61	1.52 (0.76~2.34)
	effect ^a^	−0.04(−0.07~0.08)	−0.02(−0.05~0.01)	−0.08(−0.16~0.00)	0.11(0.05~0.18)
	*p*	4.02 × 10^−1^	2.60 × 10^−1^	5.80 × 10^−2^	**1.00 × 10**^−**3**^
*KCNJ1*	CC (*n* = 1774)	4.89 ± 0.92	1.30 ± 0.31	3.06 ± 0.81	1.28 (0.96~1.74)
rs675759	CG (*n* = 514)	4.88 ± 0.93	1.30 ± 0.33	3.07 ± 0.82	1.30 (0.93~1.83)
	GG (*n* = 35)	4.90 ± 0.98	1.30 ± 0.26	2.99 ± 0.86	1.29 (1.09~1.83)
	effect ^a^	0.01 (−0.07~0.09)	0.00 (−0.03~0.03)	0.01 (−0.07~0.07)	0.02 (−0.04~0.07)
	*p*	8.83 × 10^−1^	8.73 × 10^−1^	9.31 × 10^−1^	5.85×10^−1^
*KCNJ1*	TT (*n* = 1813)	4.89 ± 0.92	1.29 ± 0.30	3.06 ± 0.81	1.28 (1.05~1.75)
rs675388	TA (*n* = 478)	4.91 ± 0.92	1.31 ± 0.33	3.08 ± 0.83	1.29 (0.92~1.83)
	AA (*n* = 32)	4.91 ± 1.04	1.28 ± 0.29	2.96 ± 0.90	1.29 (1.05~1.76)
	effect ^a^	0.04 (−0.04~0.12)	0.01 (−0.02~0.04)	0.01 (−0.06~0.08)	0.01 (−0.05~0.07)
	*p*	3.43 × 10^−1^	2.92 × 10^−1^	8.01 × 10^−1^	7.14×10^−1^
*KCNJ1*	GG (*n* = 1843)	4.90 ± 0.92	1.29 ± 0.31	3.07 ± 0.81	1.29 (0.96~1.76)
rs2846679	GA (*n* = 457)	4.84 ± 0.91	1.31 ± 0.33	3.06 ± 0.83	1.24 (0.92~1.76)
	AA (*n* = 23)	4.67 ± 0.95	1.25 ± 0.30	2.72 ± 0.73	1.28 (0.99~1.69)
	effect ^a^	−0.05 (−0.14~0.03)	0.01 (−0.02~0.04)	−0.03 (−0.10~0.05)	−0.02 (−0.08~0.04)
	*p*	2.35 × 10^−1^	3.92 × 10^−1^	4.72 × 10^−1^	5.49×10^−1^

^a^ Effects are measured as additive effects, which correspond to the average change in phenotype when the major allele is replaced by the minor allele. Adjusted for sex, age, BMI, waist circumference, smoking, drinking, and exercise. Bold values are still statistically significant after a Bonferroni correction.

**Table 4:** Cumulative effects of the risk alleles on TC, LDL-C, and TG.

Lipids	Genotype Score ^a^					Β (95% CI)	*p* for Trend
	0	1	2	3	4		
TC							
N	1150	562	473	111	27		
Mean ± SD	4.83 ± 0.89	4.81 ± 0.89	4.89 ± 0.91	4.99 ± 0.93	5.34 ± 0.85	0.085 (0.048~0.122)	**7.00 × 10^−6^**
LDL-C							
N	1150	562	473	111	27		
Mean ± SD	3.01 ± 0.77	3.06 ± 0.83	3.15 ± 0.88	3.15 ± 0.88	3.41 ± 0.79	0.075 (0.042~0.108)	**9.00 × 10^−6^**
TG							
N	1535	676	102	10	-		
Median (IQR)	1.23 (0.95~1.68)	1.31 (0.96~1.86)	1.37 (1.00~1.87)	1.31 (0.98~2.08)	-	0.096 (0.051~0.140)	2.90×10^−5^

^a^ Genotype score represents the number of risk alleles associated with TC (rs1003723 and rs6413504), LDL-C (rs1003723 and rs6413504), and TG (rs662145 and rs11643718). Adjusted for sex, age, BMI, waist circumference, smoking, drinking, and exercise. Bold values are still statistically significant after a Bonferroni correction.

## Supplementary Materials

The following are available online at https://www.mdpi.com/1660-4601/16/17/3207/s1, Table S1: Sequences of primers and probes for the PCR-LDR title.
