# Supplementary material for: Polymorphisms in PCSK9, LDLR, BCMO1, SLC12A3, and KCNJ1 Are Associated with Serum Lipid Profile in Chinese Han Population
Source: Int J Environ Res Public Health. 2019 Sep 2;16(17):3207. doi: 10.3390/ijerph16173207 (PMC6747169; doi:10.3390/ijerph16173207)
Supplement: Supplementary file 1 [file ijerph-16-03207-s001.pdf]

Polymorphisms in *PCSK9, LDLR, BCMO1, SLC12A3, KCNJ1* are associated with Serum Lipid Profile in Chinese Han Population

Zheng Li,[1,*] Tian-Yu Zhao, [1,2,*] Xiao-Hua Tan, [1]Song Lei,[1,2] Liu Huang, [1] and Lei Yang [1]

[1] Medical School, Hangzhou Normal University, Hangzhou 310000, Zhejiang, China.
[2] Medical School, Shihezi University, Shihezi 832000, Xinjiang, China.

[*] These authors contributed equally to this work.

Corresponding author: Lei Yang，Medical School, Hangzhou Normal University, 2318 Yuhangtang Rd, Hangzhou, Zhejiang, China. Tel: +86-05-712-8865-010, yanglei62@hznu.edu.cn

Supplementary Materials

Table S1. The sequences of primers and probes for PCR-LDR.

SNPs	primers	probes
rs12934922	F AAGCTACTACCACAGCTTTGG	A：GATGGCAACCGCATACATCCGGAGA
	R TTGCCTTAAACTTCACCCTCC	T：TTTGATGGCAACCGCATACATCCGGAGT
		R：-P-ATGAGCTGGGCCTCCTGCCTGGCTT-FAM-
rs2479409	F GCTCTCTTTCCCCTTTTCATC	A：TTTTTTTTTTTTGAATTCTGAATGTACCTATATGACA
	R CCAGCCTACATGCATTTCAAG	G：TTTTTTTTTTTTTTTGAATTCTGAATGTACCTATATGACG
		R：-P-TCTTTGCAAACTTAAAACCTGAATCTTTTTTTTT-FAM-
rs662145	F TGAGTGTGAAAGGTGCTGATG	C：TTTTTTTTTTTTTTTTCTAGCCAGAGGCTGGAGACAGGTGC
	R TAGAGCAGAGTAAAGGTGGCT	T：TTTTTTTTTTTTTTTTTTTCTAGCCAGAGGCTGGAGACAGGTGT
		R：-P-GCCCCTGGTGGTCACAGGCTGTGCCTTTTTTTTTTTT-FAM-
rs2738466	F ACCGAGACCAAACTCATTCAC	A：TTTTTGTCAGGACACCAGCCTGGTGCCCA
	R CTGAACTGAGAAAGTGCAAGG	G：TTTTTTTTGTCAGGACACCAGCCTGGTGCCCG
		R：-P-TCCTCCCGACCCCTACCCACTTCCATTT-FAM-
rs1003723	F AATCTACTGGTCTGACCTGTC	C：TTTTTTTTCGCAGGTGAGATGAGGGCTCCTGGC
	R TAGATGTTGCTGTGGATCCAG	T：TTTTTTTTTTTCGCAGGTGAGATGAGGGCTCCTGGT
		R：-P-GCTGATGCCCTTCTCTCCTCCTGCCTTTTTT-FAM-
rs6413504	F ACTTTGGCTTTTGCCCTGAGAG	A：TTTTTTTTTTTTGTGGCCTCCAGCCGTGTTTCCTGAA
	R TCATCCTCCAGACTGACCATC	G：TTTTTTTTTTTTTTTGTGGCCTCCAGCCGTGTTTCCTGAG
		R：-P-TGCTGGACTGATAGTTTCCGCTGTTTTTTTTTT-FAM-
rs11643718	F AGAATTGCTTGAACCTGGGAG	A：TCCCTGACATCAACCAGAACCCTCA
	R AGGCACACAGTTGGCCCTTCT	G：TTTTCCCTGACATCAACCAGAACCCTCG
		R：-P-GGCTGAGCAGTAAGTTCTGTTTTGG-FAM-

rs675759	F CTAAAAGGGACCAGCTTATGG	C：TTTTTTTTCTGGAAAAAAACTGGTACTCAAAGC
	R GGAACCTGACTCAGTTAATGC	G：TTTTTTTTTTTCTGGAAAAAAACTGGTACTCAAAGG
		R：-P-TGCTTAGGAATCAAAATGTTTTCAGTTTTTT-FAM-
rs675388	F CTAAAAGGGACCAGCTTATGG	C：TTTTTTTTTTTTTTTTTTCAGATCCCCACCTGCATCATTCC
	R GGAACCTGACTCAGTTAATGC	T：TTTTTTTTTTTTTTTTTTTTTCAGATCCCCACCTGCATCATTCT
		R：-P-GAATGTGTCCTGGAAAAAAAACTGGTACTCTTTTTTTT-FAM-
rs2846679	F CCCAGAAGATGAGTGAATTCC	A：TTTTTTTTTTTTGTCTAAGGCAGGAGGGAAAAGGAAA
	R ACTTCCCTGGGTCTTGAGAAG	G：TTTTTTTTTTTTTTTGTCTAAGGCAGGAGGGAAAAGGAAG
		R：-P-GAAGGTCTGGGGGAAAGGCTGGCCCTTTTTTTTT-FAM-
